# Prevalence, Intensity and Associated Factors of *Cysticercus tenuicollis* in Small Ruminants in the Northwest Region of Cameroon

**DOI:** 10.1002/vms3.70307

**Published:** 2025-03-27

**Authors:** Prudentia Yensi Lawan, Aziwo Tatanja Niba, Julius Awah‐Ndukum

**Affiliations:** ^1^ Divisional Delegation of Livestock Fisheries and Animal Industries Mezam Bamenda Northwest Region Bamenda Cameroon; ^2^ Department of Animal Production Technology College of Technology The University of Bamenda Bamenda Cameroon

**Keywords:** associated factors, *Cysticercus tenuicollis*, Northwest Cameroon, prevalence, sheep and goats

## Abstract

**Background:**

*Cysticercus tenuicollis* infection, which can cause production and economic losses in livestock, is neglected in most African countries, including Cameroon.

**Objective:**

To determine the prevalence, intensity and associated factors of *C. tenuicollis* in small ruminants in the Northwest region, Cameroon.

**Materials and Methods:**

A total of 1106 small ruminants (493 sheep; 613 goats) originating from divisions of the study region and destined for slaughter in Bamenda municipality were examined. Following slaughter, intensive meat inspections were performed to detect *C. tenuicollis* cysts based on standard procedures.

**Results:**

Overall, the prevalence of *C. tenuicollis* was 34.36% (31.62%–37.21%), and no difference (*χ* ^2^ = 1.43, *p* = 0.23) was observed between goats (35.89% [32.19%–39.76%]) and sheep (32.45% [28.47%–36.70%]). *C. tenuicollis* cyst was prevalent in all divisions in the region and detected during the entire study period. Weight, body condition score, pregnancy and lactating status of females, origin of the animals and season were the major (*p* < 0.05) factors in goats and only age (*p* < 0.05) in sheep. *C. tenuicollis* cysts were predominant in the abdominal cavity (97.90%) (OR = 2477.79; 889.45–6902.46; *p* < 0.0001, *χ* ^2^ = 701.19) and mainly attached to the omentum (71.84%) (OR = 20.03; 13.53–29.66; *p* < 0.0001, *χ* ^2^ = 269.13) compared to the pelvic cavity and other organs, respectively.

**Conclusion:**

The study showed high prevalence and widespread distribution of *C. tenuicollis* infection in small ruminants and suggested that cysticercosis in small ruminants and its associated socio‐economic implications for livestock production are neglected in Northwest Cameroon. Concerted veterinary–livestock farmer efforts, monitoring of infected small ruminant populations and regular parasite control in dogs in contact with small ruminants and prevention of contamination of pastures with *T. hydatigena* eggs by barring access of potential definitive hosts are essential for the control of the disease.

## Graphical abstract

*Cysticercus tenuicollis* infection, which can cause production and economic losses in livestock, is neglected in most African countries, including Cameroon, where there are poor sanitary conditions and animal husbandry with dogs in the same environment. There are growing concerns on the epidemiology of *Taenia hydatigena* cysticercosis in livestock in many endemic areas. This study, carried out from October 2022 to September 2023, reported high prevalence, widespread distribution and associated factors of *C. tenuicollis* infection in sheep and goats in Northwest Cameroon.

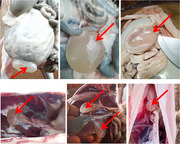

## 1 Introduction

Taeniasis/cysticercosis caused by *Taenia hydatigena* is a parasitic disease transmitted between canids and livestock, including ruminants and pigs (Gessese et al. 2014; Miran et al. 2017; Khouloud et al. 2019). The disease is worldwide in distribution and particularly endemic in most of Africa, where there are poor sanitary conditions, free‐range animal husbandry, stray and scavenging dogs, lack of awareness, contact between wild canids and livestock and inadequate control of animal diseases (Mekuria et al. 2013; Nyero et al. 2015; Djonmaïla 2016; Awe 2017; Djiatche 2017; Miran et al. 2017). *T. hydatigena* is an adult parasite that lodges in the intestines of domestic and wild canids, with the metacestode (*Cysticercus tenuicollis*) stage residing in ruminants and pigs where they are reared under free‐range systems with *T. hydatigena*‐infected canids in the same environment (Bayu et al. 2013; Khouloud et al. 2019). *C. tenuicollis* infection is responsible for production losses and mortality in livestock (Wondimu et al. 2011; Al‐Sudani and Al‐Amery 2022), causing huge economic losses due to condemnation of infected offal and meat (Gessese et al. 2014; Scala et al. 2015; Miran et al. 2017; Torgerson et al. 2019).

Migration of the cysticerci through organs can lead to formation of haemorrhagic and fibrotic tracts, serofibrinous peritonitis in the liver (Samuel and Zewde 2010; Oryan et al. 2012; Scala et al. 2015), traumatic hepatitis and death in young lambs (Corda et al. 2020; Mohammed and Kadir 2020). Diagnosis of cysticercosis in livestock is usually based on the demonstration of the metacestode attached to organs during meat inspection and necropsy (WHO/FAO/OIE 2005; OIE 2008). The *C. tenuicollis* metacestode predominantly attaches to the omentum, mesentery and occasionally on the liver surface, lungs, kidneys, brain, ovaries, uterine tubes, cervix and vagina (WHO/FAO/OIE 2005; Singh et al. 2015; Dyab et al. 2017; Ensieh et al. 2020). Though the adult parasites are not highly pathogenic for the definitive hosts, haemorrhagic tracks, traumatic hepatitis and death of young animals may occur following migration of cysticerci in the liver of intermediate hosts (Oryan et al. 2012; Corda et al. 2020).

The metacestode infection due to *C. tenuicollis* is usually a neglected cysticercosis with grossly underestimated animal health and economic impacts, particularly in endemic areas of Asia and Africa (Wondimu et al. 2011; Oryan et al. 2012; Mokhtaria et al. 2018; Mohammed and Kadir 2020). In communities where there are stray and scavenging dogs, poor housing of dogs and irresponsible dog ownership and interactions of dogs and livestock, eggs of the tapeworm are released with the dog faeces in the environment (Awah‐Ndukum et al. 2004; Asmare et al. 2016; Al‐Sudani and Al‐Amery 2022). Following ingestion by animals, the eggs hatch and oncospheres develop in the animal tissues, causing cysticercosis (Yalelet et al. 2018). Thus, in areas where *T. hydatigena*‐infected canids and ruminants (domestic and wild) live in close association and share the same environment (Khouloud et al. 2019), *C. tenuicollis* metacestodes will develop in the ruminants (Bakhraibah and Alsulami 2018; Corda et al. 2020).

The prevalence of *C. tenuicollis* was 0.45% in goats in Saudi Arabia (Bakhraibah and Alsulami 2018) and 56.8% in sheep in Central Oromia, Ethiopia (Wondimu et al. 2011); 4.83% in sheep and goats in Northern India (Singh et al. 2015); 72.38% in goats in Dessie, Ethiopia (Gessese et al. 2014) and 19.2% (15.1%–24.1%) in pigs in Northern Cameroon (Assana et al. 2019). Also, eggs of *T. hydatigena* predominantly released by scavenging domestic dogs (Djiatche 2017; Al‐Sudani and Al‐Amery 2022) infect sheep, goats, pigs and cattle in areas where livestock and stray dogs interact and share the same environment (Assana et al. 2010; Djonmaïla 2016; Awe 2017).

Though *T. hydatigena* is non‐zoonotic, cysticercosis can cause great losses to socio‐cultural activities and livelihoods of agropastoral communities that depend on animal husbandry (ruminants, pigs). There are growing concerns about the epidemiology and control of *T. hydatigena* cysticercosis in domestic ruminants, including sheep and goat, in endemic areas, including Africa. However, data on the epidemiology and impacts of *T. hydatigena* taeniosis/cysticercosis in sheep and goat production systems in Cameroon is scanty. The level of awareness of these livestock handlers of the disease, such as its impacts on animal health and the livelihoods (socio‐economic, cultural and religious activities) of agropastoral communities in the country, is not known. In this context, this study was carried out to estimate the prevalence, intensity and associated risk factors of *C. tenuicollis* in small ruminants in the Northwest region of Cameroon.

## 2 Materials and Methods

### 2.1 Study Animals and Areas

**FIGURE 1:**
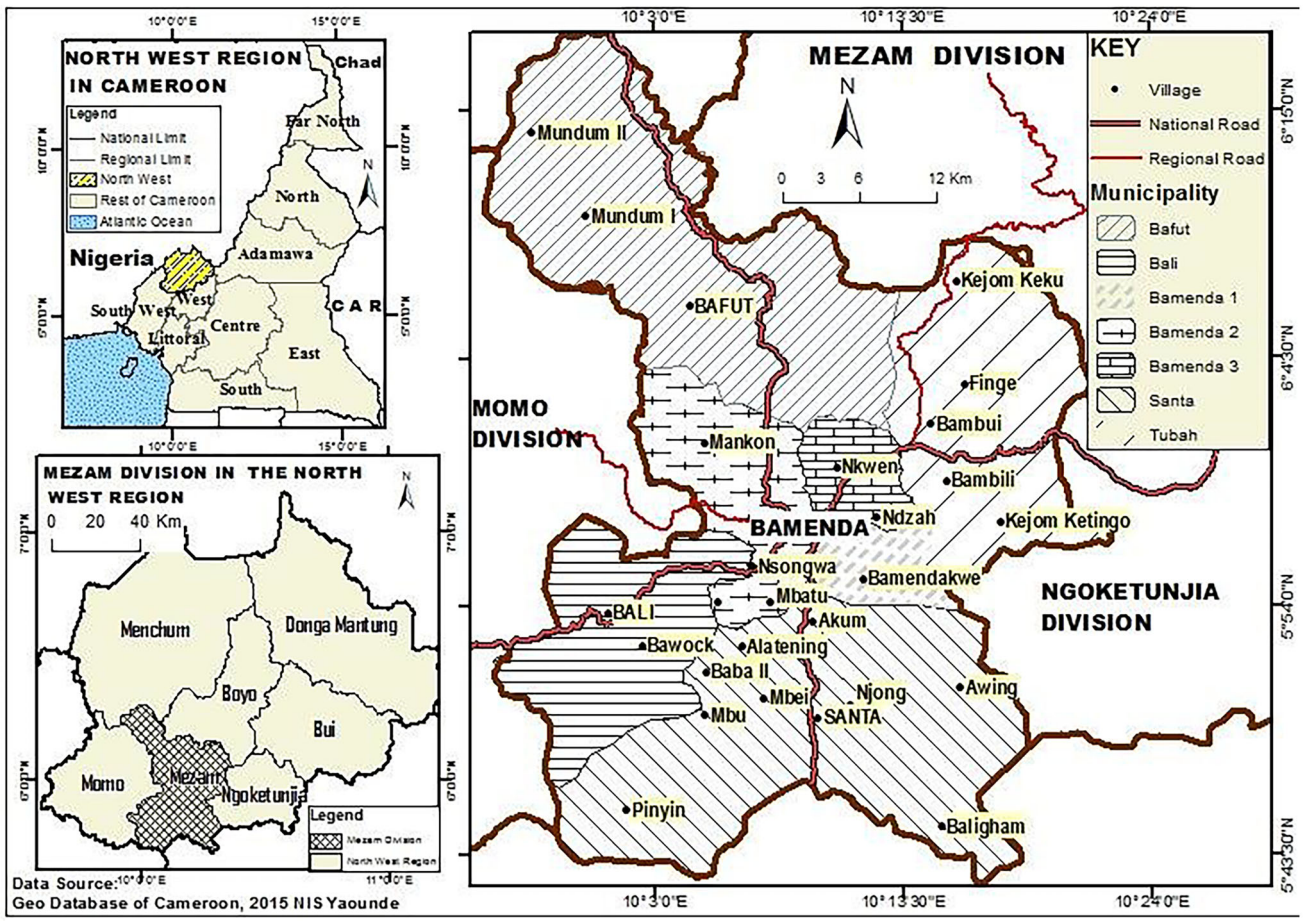
Map of Cameroon showing the administrative divisions (7) in the Northwest region and sites for small ruminant markets and slaughter slabs in study areas (Bambui, Nkwen and Mankon areas) of Mezam division. *Source*: Adapted from Nis (2015).

Sheep and goats from local livestock markets in Bamenda municipality originating from the administrative divisions (07) of the Northwest region of Cameroon (5°45″–9°9″ N and 9°13″–11°13″ E), destined for slaughter, during the period October 2022 to September 2023, were sampled for the study (Figure 1). The Northwest region of Cameroon is located within an altitude of 500–3000 m above sea level, and it is characterized by fertile volcanic soils and colder temperatures (21°C on annual average but with distinct differences according to altitude). The region has two main seasons: the rainy season starts from March to October, and the dry season starts from November to February. The choice of the study areas was due to the following: (1) The Northwest region is ranked as a major livestock production area, including sheep and goats, in the country (MINEPIA 2021). The ethnic groups in the study area are mostly agropastoralists with passionate traditions for livestock rearing. (2) There is an abundance of stray and scavenging dogs that share the same environment with livestock (Awah‐Ndukum 2003; Awah‐Ndukum et al. 2004), and there is no documented evidence or study on the status of *C. tenuicollis* in small ruminants in the Northwest region of Cameroon. It was common to find mixed‐livestock husbandry, several domestic species (cattle, sheep, goats, horses, donkeys and fowls) and domestic dogs (owned and stray) cohabiting within the same farm or being present in the same microenvironment (such as livestock markets, communal pastures, watering points, mineral lick points and vaccination posts). (3) Cysticerci have been detected in slaughtered livestock, especially in pigs (Njila et al. 2003) and domestic ruminants (cattle, sheep and goats) (MINEPIA, personal communication); and there are many communities with strong cultures of livestock rearing for livelihood in the region.

### 2.2 Animal Population and Selection for the Study

A default expected prevalence of 50% was used to determine the sample size required to estimate the prevalence of cysticercoid cases at meat inspection in domestic small ruminants (sheep and goats) with a desired 95% confidence and precision of ≥ 5% using the following formula: $n=\frac{1.96^{2}P_{\exp}\left(1-P_{\exp}\right)}{d^{2}}$ where; *n* = required sample size; *P*_exp_ = expected prevalence; and *d* = desired absolute precision (Thrusfield 2007).

Small ruminants (sheep and goats) originating from all the seven administrative divisions (corresponding to 25 administrative subdivisions) in the Northwest region of Cameroon and imported into the small ruminant markets in the Bamenda municipality were targeted in the study. However, traders and/or butchers present in the markets were listed during weekly visits, and all animals that were destined for slaughter and owned by willing trader–butchers who gave their verbal informed consent were included in the study. These trader–butchers and their animals (irrespective of the number) were accompanied by the researcher to slaughter slabs for general and intensified inspections, including post‐mortem examination and harvesting of cysts in infected carcasses. Overall, 1106 slaughtered small ruminants (493 sheep and 613 goats) judged as fit for slaughter during ante‐mortem inspection were used in this study.

Information relating to location, husbandry practices, breed, physiological status, sex, age and origin of the animals provided by the handlers was recorded. In addition, the breeds were determined by phenotypic description (Meutchieye et al. 2014; Simo and Meutchieye 2015); age by dental examination (Erbeto et al. 2010) and body condition score (BCS) by ranking on a scale of 1 to 5 (Villaquiran et al. 2012) and classified as *lean* (1 to 2), *moderate* (3 to 4) and *fat* (5) (Kassa et al. 2012). The small ruminants used in this study were reared traditionally with or without shelter, such as free‐range (scavenging) or extensive systems and semi‐intensive that depend on natural pasture in agropastoral communities where there are interactions between small ruminants and stray and scavenging dogs.

### 2.3 Detection of *Cysticercus tenuicollis* in Sheep and Goats

Following the slaughter of the selected small ruminants (sheep and goats) in this study, intensive meat inspections were carried out by PYL assisted by the veterinary staff at the slaughter slabs based on the government's legislation regulating veterinary health inspection and notification of contagious animal diseases (MINEPIA 2000). In addition, evidence of pathologies was supported by post‐mortem examination of carcasses as previously described (FAO 1994; MINEPIA 2000; Grist 2011), and the inspection procedure employed systematic visual examination and palpation of all visceral organs in the thoracic, abdominal and pelvic cavities for bladder‐like cysts (Asmare et al. 2016; Yigizaw et al. 2017). A transparent bladder‐like structure containing a clear fluid and a long neck with white corn‐sized spots in the fluid was detected as *C. tenuicollis* vesicle (Rostami et al. 2015; Singh et al. 2015; Hailu et al. 2019; Khouloud et al. 2019; Felefl and Laban 2020). Infected organs and the site of localization of the cyst(s) and, where possible, the parasite migration routes on organs were noted in the pre‐prepared data collection sheets. The prevalence and intensity of infection were obtained by calculating the rate of infested animals among the examined animals and counting the number of cysts per infested animal, respectively (Thrusfield 2007, Al‐Hamzawi and Al‐Mayali 2020).

### 2.4 Data Analysis

All obtained data were initially entered into Excel 2010 and then transferred to SPSS 20 for further analysis. The Chi‐square test was used to test significant levels (Fisher test where observations were less than 5) within factors on prevalence rates; odds ratios and regression analysis were used to assess the strength of association of different factors with the prevalence of *C. tenuicollis* in small ruminants along 95% confidence intervals and statistical significance set at *p* < 0.05 (Thrusfield 2007).

## 3 Results

**FIGURE 2:**
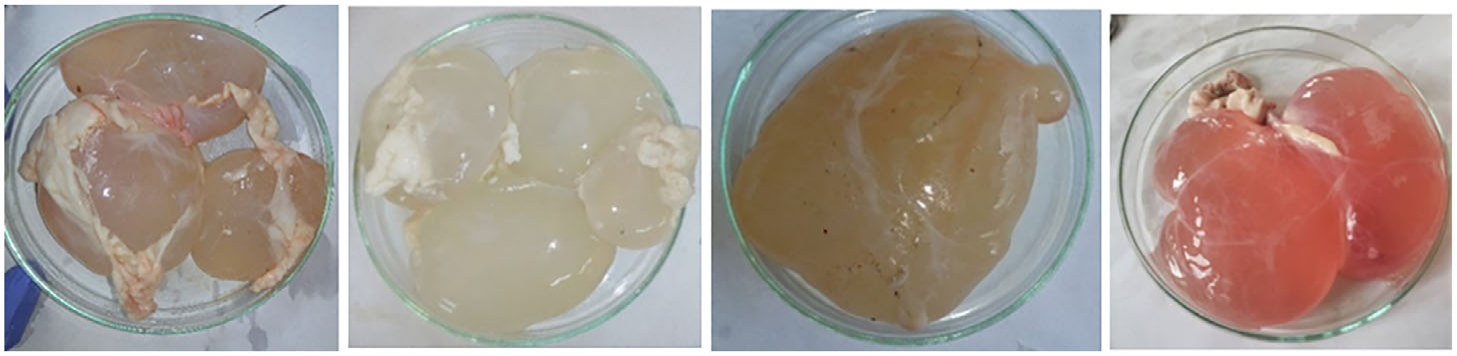
Cysticercus tenuicollis isolated from slaughtered sheep and goat in the Northwest region of Cameroon.

The sheep and goats slaughtered in the Bamenda municipality were mainly of the Djallonke type. The results showed that sheep and goats harboured varied larval stages of *C. tenuicollis* (Figure 2). The infection was widespread in the study region, and the *C. tenuicollis* metacestode was detected during the entire study period.

The prevalence of *C. tenuicollis* according to sex, age, weight, BSC, physiological status of female animals, origin of the animal and season are shown in Table 1. Overall, 380/1106 (34.36%; 95% CI: 31.62–37.21) animals sampled were infected with the metacestode *C. tenuicollis*, with 220/613 (35.89%; 95% CI: 32.19–39.76) being goats and 160/493 (32.45%; 95% CI: 28.47–36.70) being sheep. Though there was no difference (*χ* ^2^ = 1.43, *p* = 0.23) between the proportion of infected goats and sheep, the prevalence of infected animals (sheep and goats) showed distinct (*p* < 0.05) variations between seasons and according to the weight, physiological (pregnant or lactating) status and location of origin of the animals in study sites (divisions). However, the prevalence rate was significantly (*p* < 0.05) influenced by location of origin, weight, BSC and physiological (pregnant or lactating) status of the females as well as season for goats and only age for sheep (Table 1).

**TABLE 1:** Prevalence of *Cysticercus tenuicollis* in sheep and goats in Northwest region (NW) of Cameroon.

		Goats			Sheep			Total		
Parameter	Variable	Number examined	Prevalence, % (95% CI)	*p* (*χ* ^2^)	Number examined	Prevalence, % (95% CI)	*p* (*χ* ^2^)	Number examined	Prevalence, % (95% CI)	*p* (*χ* ^2^)
Sex	Female	396	36.87 (32.27–41.86)	0.49 (0.47)	246	34.96 (29.27–41.11)	1.4 (0.23)	642	36.14 (32.52–39.93)	0.14 (2.15)
	Male	217	34.10 (28.12–40.63)		247	29.96 (24.59–35.94)		464	31.90 (27.82–36.27)	
Age (years)	Age ≤ 1	30	44.44 (29.54–60.41)	0.15 (6.71)	52	19.23 (10.80–31.90	0.03 (10.82)	82	31.71 (22.65–42.41)	0.71 (2.12)
	1 < age ≤ 2	177	32.77 (26.29–39.99)		162	35.19 (28.26–42.81)		339	33.92 (29.09–39.11)	
	2 < age ≤ 3	227	37.00 (30.98–43.45)		177	28.25 (22.13–35.29)		404	33.17 (28.76–37.90)	
	3 < age ≤ 4	132	31.82 (24.48–40.18)		88	43.18 (33.33–53.60)		220	36.36 (30.29–42.90)	
	Age > 4	47	42.55 (29.51–56.71)		14	35.71 (16.34–61.23)		61	40.98 (29.53–53.50)	
Weight (W) of animal (kg)	W ≤ 20	68	44.12 (32.95–55.92)	0.02 (11.70)	20	25.00 (11.19–46.87)	0.39 (4.07)	88	39.77 (30.18–50.22)	0.01 (13.68)
	20 < W ≤ 30	165	30.91 (24.36–38.33)		121	23.97 (17.24–32.30)		286	27.97 (23.09–33.62)	
	30 < W ≤ 40	249	40.96 (35.03–47.16)		256	32.42 (26.98–38.38)		505	38.61 (34.46–42.93)	
	40 < W ≤ 50	83	32.53 (23.42–43.19)		70	35.71 (25.50–47.41)		153	33.99 (26.96–41.18)	
	W > 50	48	20.83 (11.73–34.26)		26	30.77 (16.50–49.99)		74	24.32 (15.97–35.20)	
Body condition score	Fat	143	27.27 (20.64–35.06)	0.04 (6.49)	147	38.10 (30.65–46.16)	0.22 (3.06)	290	32.76 (27.61–38.36)	0.77 (0.52)
	Medium	385	39.22 (34.47–44.18)		304	29.93 (25.06–35.30)		689	35.12 (31.65–38.76)	
	Thin	85	35.29 (25.97–45.88)		42	30.95 (19.07–46.02)		127	33.86 (26.21–42.46)	
Physiological status of female animals	Pregnant or lactating	184	50.00 (42.85–57.15)	0.0006 (11.69)	140	37.14 (29.58–45.39)	0.70 (0.15)	324	44.44 (39.13–49.88)	0.002 (9.22)
	Not pregnant and not lactating	212	25.47 (20.08–31.74)		106	32.08 (23.95–41.46)		318	27.67 (23.04– 32.83)	
Origin of animal (administrative division in NW region)	Boyo	108	25.00 (17.79–33.93)	0.001 23.15	77	28.57 (19.69–39.49)	0.36 (6.12)	185	26.49 (20.66–33.28)	0.004 (19.10)
	Bui	170	41.76 (34.61–49.28)		44	47.73 (33.76–62.07)		214	42.99 (36.54–49.69)	
	Donga‐Mantung	131	40.46 (32.44–49.02)		21	33.33 (17.19–54.62)		152	39.47 (32.05–47.41)	
	Menchum	18	50.00 (29.03–70.97)		18	33.33 (16.28–56.25)		36	41.67 (27.14–57.80)	
	Mezam	80	20.00 (12.70–30.05)		111	32.43 (24.44–41.60)		191	27.23 (24.14–33.95)	
	Momo	82	45.12 (34.81–55.87)		216	30.09 (24.37–36.51)		298	34.23 (29.07–39.79)	
	Ngoketunjia	24	29.17 (14.92–49.17)		6	50.00 (18.76–81.24)		30	33.33 (19.23–51.22)	
Season	Rainy season	459	38.99 (34.79–43.36)	< 0.0001 (30.12)	368	32.88 (28.28–37.83)	0.73 (0.12)	827	37.97 (34.73–41.33)	<0.0001 (18.98)
	Dry season	154	17.53 (12.34–24.30)		125	31.20 (23.74–39.78)		279	23.66 (19.05–28.98)	
**Overall**		613	35.89 (32.19–39.76)		493	32.45 (28.47–36.70)		1106	34.36 (31.62–37.21)	

The lowest and highest prevalence rates were observed in the Boyo (26.49%) and Bui (42.99%) divisions, respectively. Animals originating from Bui (OR = 1.92 (95% CI: 1.26–2.93); *χ* ^2^ = 9.33, *p* = 0.002) and Donga‐Mantung (OR = 1.81 (95% CI: 1.14–2.87); *χ* ^2^ = 6.43, *p* = 0.01) divisions were more likely to be infected with the metacestode compared to animals originating from Boyo division (Table 2). Also, infection rates were significantly higher in pregnant or lactating animals (OR = 1.96 (95% CI: 1.33–2.90); *χ* ^2^ = 11.69, *p* = 0.0006) and animals sampled during the rainy season (OR = 1.97 (95% CI: 1.45–2.69); *χ* ^2^ = 18.95, *p* < 0.0001) compared to non‐pregnant and non‐lactating animals and animals sampled in the dry season, respectively.

**TABLE 2:** Assessment of potential risk factors of *Cysticercus tenuicollis* in sheep and goats in Northwest region (NW) of Cameroon.

		Goats			Sheep			Total		
Parameter	Variable	Prevalence (%)	Odds ratio (95% CI)	*p* (*χ* ^2^)	Prevalence (%)	Odds ratio (95% CI)	*p* (*χ* ^2^)	Prevalence (%)	Odds ratio (95% CI)	*p* (*χ* ^2^)
Sex	Female	36.87	1.13 (0.80– 1.60)	0.47 (0.49)	34.96	1.26 (0.86–1.83)	0.24 (1.41)	36.14	1.21 (0.94–1.56)	0.14 (2.15)
	Male	34.10	1	—	29.96	1	—	31.90	1	
Age (years)	Age ≤ 1	44.44	2.45 (1.09–5.48)	0.03 (4.92)	19.23	1	—	31.71	1	
	1 < age ≤ 2	32.77	1.04 (0.64–1.69)	0.86 (0.03)	35.19	2.28 (1.06–4.88)	0.03 (4.66)	33.92	1.11 (0.66–1.85)	0.70 (0.15)
	2 < age ≤ 3	37.00	1.26 (0.80–1.98)	0.32 (0.99)	28.25	1.65 (0.77–3.55)	0.19 (1.69)	33.17	1.07 (0.64–1.78)	0.79 (0.07)
	3 < age ≤ 4	31.82	1	—	43.18	3.19 (1.4–7.16)	0.004 (8.32)	36.36	1.23 (0.72–2.11)	0.45 (0.57)
	Age > 4	42.55	1.59 (0.80–3.15)	0.18 (1.76)	35.71	2.33 (0.64–8.50)	0.28^a^	40.98	1.51 (0.75–2.98)	0.25 (1.31)
Weight (W) of animal (kg)	W ≤ 20	44.12	3.00 (1.29–6.98)	0.01 (6.75)	25.00	1.06 (0.35–3.16)	1^a^	39.77	2.05 (1.04–4.06)	0.04 (4.36)
	20 < W ≤ 30	30.91	1.70 (0.79–3.67)	0.17 (1.85)	23.97	1		27.97	1.21 (0.67–2.18)	0.53 (0.39)
	30 < W ≤ 40	40.96	2.64 (1.26–5.53)	0.01 (6.94)	32.42	1.52 (0.93–2.49)	0.09 (2.81)	38.61	1.96 (1.12–3.43)	0.02 (5.67)
	40 < W ≤ 50	32.53	1.83 (0.79–4.22)	0.15 (2.05)	35.71	1.76 (0.93–3.35)	0.08 (3.02)	33.99	1.60 (0.86–3.00)	0.14 (2.18)
	W > 50	20.83	1		30.77	1.41 (0.56–3.58)	0.47 (0.53)	24.32	1	
Body condition score	Fat	27.27	1		38.10	1.44 (0.95–2.18)	0.08 (3.00)	32.76	1	
	Medium	39.22	1.72 (1.13–2.62)	0.01 (6.46)	29.93	1		35.12	1.11 (0.83–1.49)	0.48 (0.51)
	Thin	35.29	1.45 (0.82–2.59	0.20 (1.63)	30.95	1.05 (0.52–2.11)	0.89 (0.02)	33.86	105 (0.68–1.63)	0.82 (0.05)
Physiological status of female animals	Pregnant or lactating	50.00	1.96 (1.33–2.90)	0.0006 (11.69)	37.14	1.25 (0.73–2.13)	0.68 (0.41)	44.44	1.61 (1.18–2.18)	0.002 (9.22)
	Not pregnant and not lactating	25.47	1		32.08	1		27.67	1	
Origin of animal (administrative division in NW region)	Boyo	25.00	1.33 (0.66–2.68)	0.42 (0.65)	28.57	1		26.49	1	
	Bui	41.76	2.87 (1.53–5.37)	0.001 (11.36)	47.73	2.28 (1.06–4.93)	0.03 (4.49)	42.99	1.92 (1.26–2.93)	0.002 (9.33)
	Donga‐Mantung	40.46	2.72 (1.42–5.20)	0.002 (9.45)	33.33	1.25 (0.44–3.51)	0.67 (0.18)	39.47	1.81 (1.14–2.87)	0.01 (6.43)
	Menchum	50.00	4.00 (1.37–11.71)	0.015^a^	33.33	1.25 (0.42–3.75)	0.69 (0.16)	41.67	1.98 (0.95–4.15)	0.07 (3.38)
	Mezam	20.00	1		32.43	1.20 (0.64–2.26)	0.57 (0.32)	27.23	1.04 (0.65–1.64)	0.86 (0.03)
	Momo	45.12	3.22 (1.60–6.47)	0.001 (11.21)	30.09	1.08 (0.61–1.91)	0.81 (0.06)	34.23	1.44 (0.96–2.17)	0.07 (3.18)
	Ngoketunjia	29.17	1.65 (0.58–4.64)	0.34 (0.90)	50.00	2.50 (0.47–13.35)	0.36^a^	33.33	1.39 (0.61–3.17)	0.43 (0.61)
Season	Rainy season	38.99	3.41 (2.16–5.38)	< 0.0001 (30.12)	32.88	1.17 (0.76–1.80)	0.47 (0.50)	37.97	1.97 (1.45–2.69)	< 0.0001 (18.95)
	Dry season	17.53	1		31.20	1		23.66	1	

^a^Fisher Exact Probability test, two tailed.

Although the overall prevalence of *C. tenuicollis* in the sample animals (sheep and goats) was significantly higher in animals that weighed ≤ 20 kg (OR = 2.05 (95% CI: 1.04–4.06); *χ* ^2^ = 4.36, *p* = 0.04) and between 30 and 40 kg (OR = 1.96 (95% CI: 1.12–3.43); *χ* ^2^ = 5.67, *p* = 0.02) than the other weight groups in the present study, variations in weights (weights ≤ 30 kg against > 30 kg) on their own seem to pose little or similar risks (OR = 1.18 (95% CI: 0.92–1.51); *χ* ^2^ = 3.26, *p* = 0.07). Detection of the parasite in small ruminants was also not influenced by the difference in age (age ≤ 2 years [young] against > 2 years [adult]) (OR = 1.06 (95% CI: 0.82–1.37); *χ* ^2^ = 0.23, *p* = 0.63).

### 3.1 Prevalence (%) of *Cysticercus tenuicollis* in Sheep and Goats According to Origin of Animals in Northwest Region of Cameroon

**FIGURE 3:**
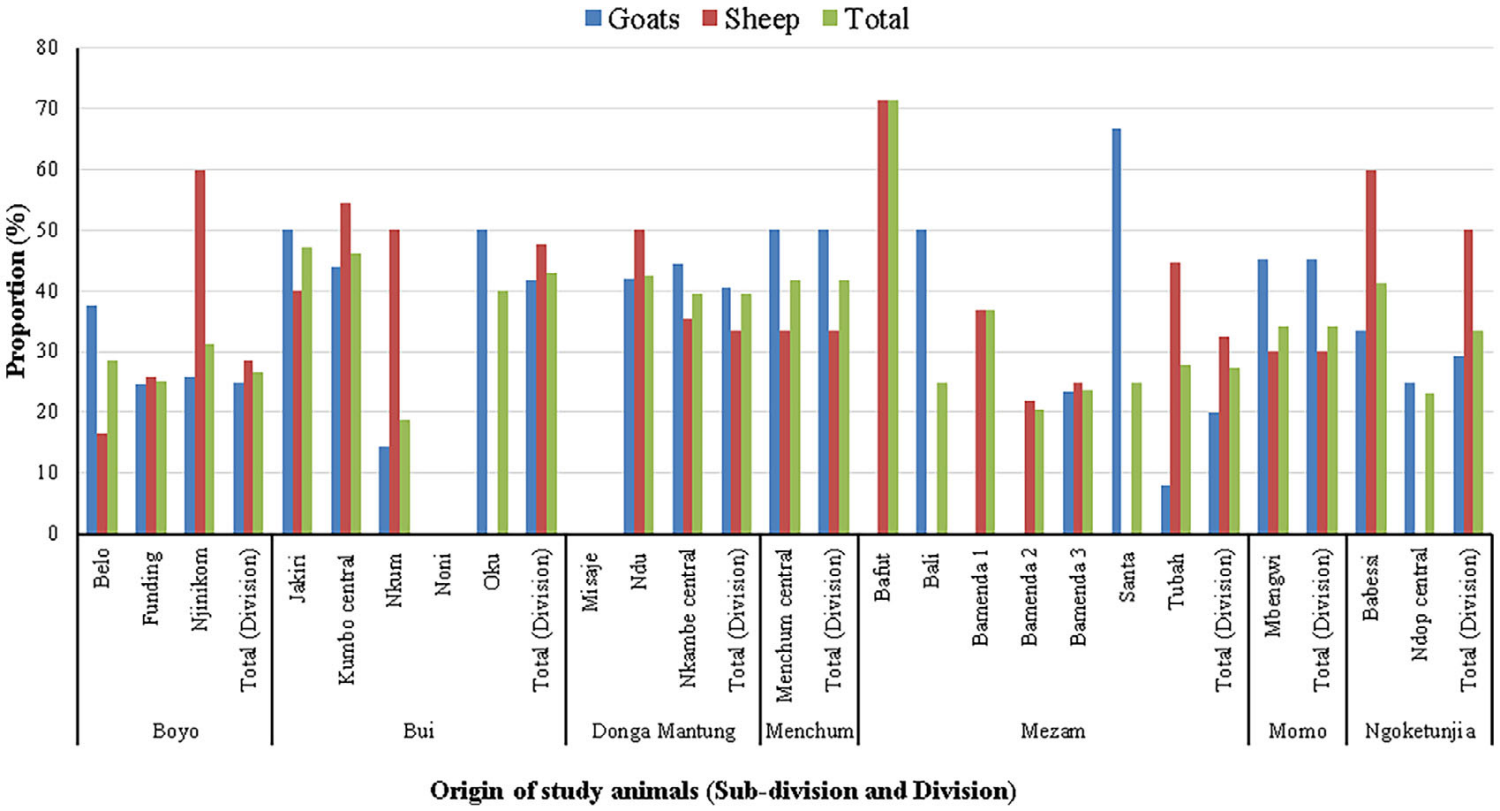
Prevalence (%) of *Cysticercus tenuicollis* in sheep and goats according to location of origin of the animal in Northwest region of Cameroon.

For the location of origin of the animals based on the sub‐division in study divisions, goats from Belo in Boyo division; Jakiri, Kumbo central and Oku in Bui division; Ndu and Nkambe central in Donga‐Manung division; Menchum central in Menchum division; Bali and Santa in Mezam division; and Mbengwi in Momo division; Babessi in Ngoketunjia division showed significantly high infection rates compared to goats from other sub‐divisions in the respective divisions (Figure 3). Contrary, sheep from Njinikom in Boyo division; Jakiri, Kumbo central and Nkum in Bui division; Ndu and Nkambe central in Donga‐Manung division; Menchum central in Menchum division; Bafut, Bamenda 1 and Tubah in Mezam division; Mbengwi in Momo division; and Babessi in Ngoketunjia division showed significantly high infection rates compared to sheep from other sub‐divisions in the respective divisions (Figure 3). The high prevalence of *C. tenuicollis* in goats was observed in animals from Santa the sub‐division (66.67%), Jakiri, Oku, Menchum central and Bali (50.00% each) and the Mbengwi sub‐division (45.12%).

While a high prevalence of the parasite in sheep was obtained in animals from the Bafut sub‐division (71.43%), Njinikom and Babessi (60.00% each) and Kumbo central sub‐division (54.55%).

### 3.2 Monthly Prevalence (%) of *Cysticercus tenuicollis* in Sheep and Goats in Northwest Region of Cameroon

**FIGURE 4:**
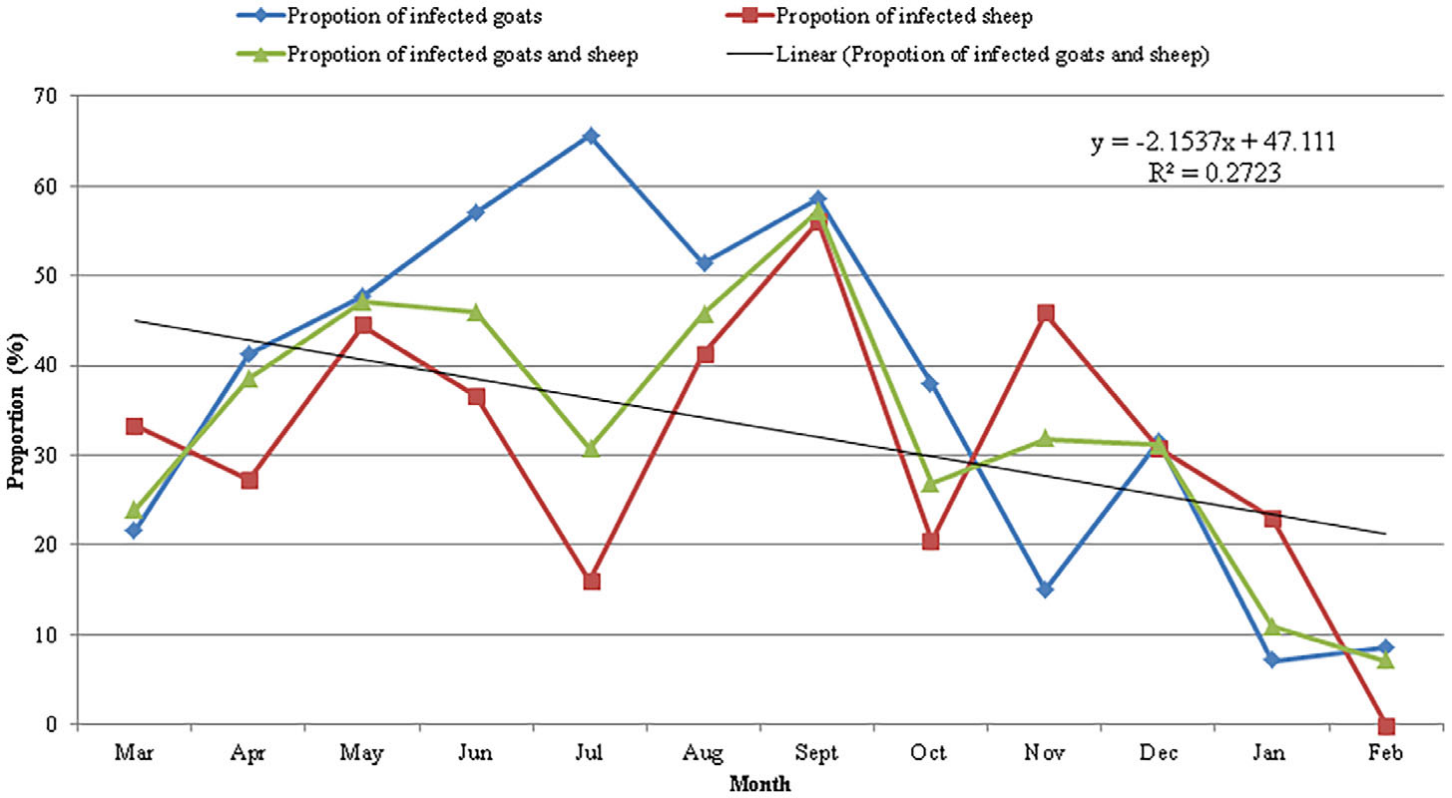
Monthly prevalence (%) of *Cysticercus tenuicollis* infection in sheep and goats in Northwest region of Cameroon.

*C. tenuicollis* infections were recorded in sheep and goats throughout the study period (Figure 4) with monthly prevalence ranging from 7.14% to 57.32% for both sheep and goats, 0% to 56.10% for sheep and 7.14% to 65.63% for goats. Overall, the detection rates of the parasite varied widely between seasons, and there was a significant difference (*p* < 0.05) between the detection rate for the wet season (37.97%; 95% CI: 34.73–41.33) and the dry season (23.66%; 95% CI: 19.05–28.98) with several fluctuating peaks observed, particularly during the months of May, July, September and December (Figure 4). Furthermore, regression modelling for the overall infection recorded during the study period revealed a slope with a negative gradient (*Y* = −2.1537*X* + 47.111; *R* ^2^ = 0.2723), suggesting a decrease in trend (from May to February) in detection rates of *C. tenuicollis* in slaughtered sheep and goats in the Bamenda municipality.

### 3.3 Distribution and Intensity of *Cysticercus tenuicollis* According to Body Cavity and Organ Sites of Infected Sheep and Goats in Northwest Region of Cameroon

**FIGURE 5:**
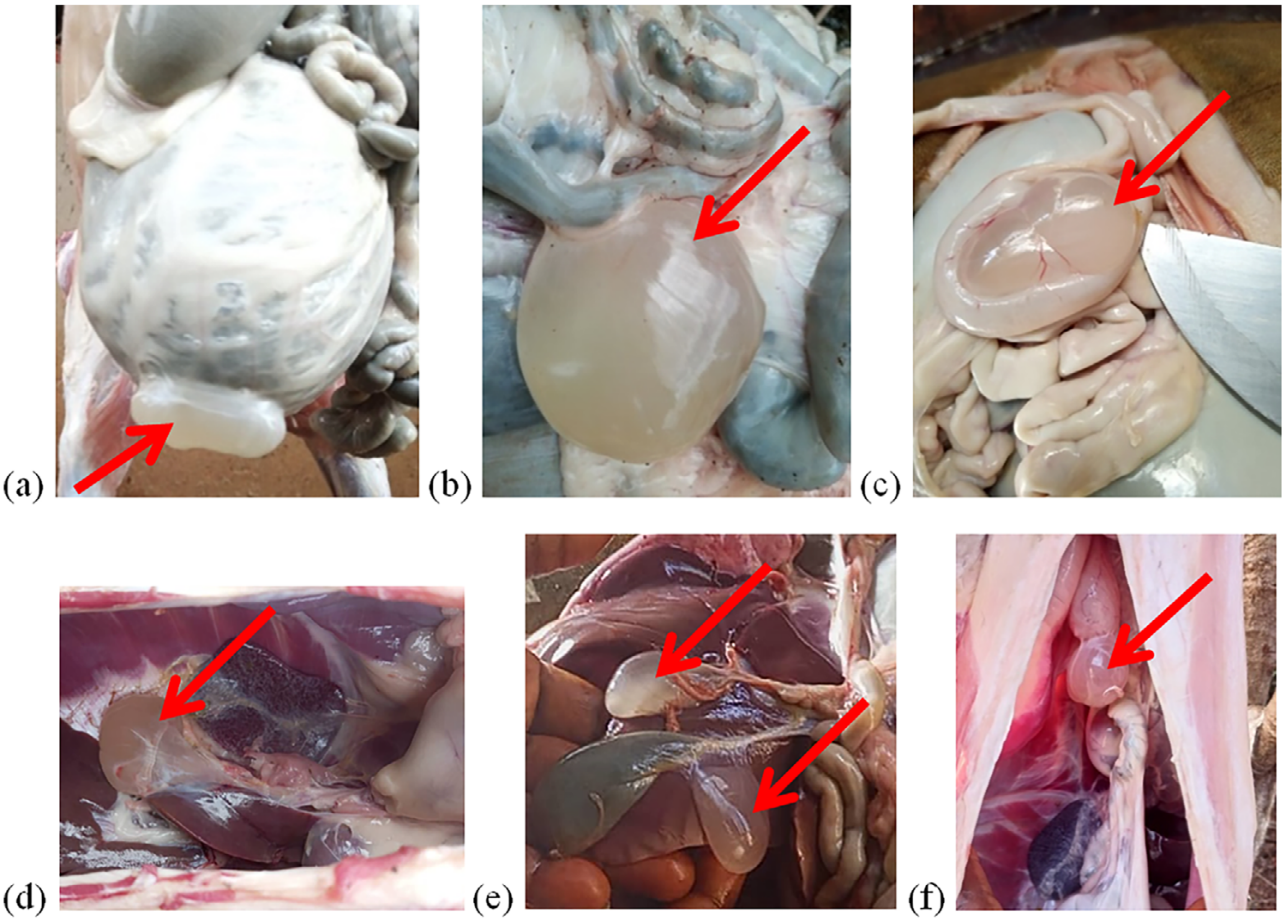
The metacestode *Cysticercus tenuicollis* attached to the (a) omentum of Sheep, (b) small intestine of goat, (c) small intestine of sheep, (d) liver of goat, (e) liver and gall bladder of sheep and (f) urinary bladder of sheep at the small ruminant slaughter slabs in the Bamenda, Northwest Cameroon.

The metacestode *C. tenuicollis* was found in the abdominal and pelvic cavities and attached to different organs of goat and sheep (Figure 5).

The distribution of *C. tenuicollis* on different organs of the goat and sheep is summarized in Table 3. The results show significant differences in the distribution of *C. tenuicollis* based on body cavities as well as attachment to a single organ or multiple organ combinations in infected animals.

**TABLE 3:** Distribution and intensity of *Cysticercus tenuicollis* according to body cavity and organ sites of infected sheep and goats in Northwest region of Cameroon.

		Goats (*N* = 220)			Sheep (*N* = 160)			Total (*N* = 380)		
Parameter	Variable	No. of cysts detected	No. of infected animals (%)	*p* (*χ* ^2^)	No. of cysts detected	No. of infected animals (%)	*p* (*χ* ^2^)	No. of cysts detected	No. of infected animals (%)	*p* (*χ* ^2^)
Body cavity of infected animals (*N* = 380)	Pelvic	5	4 (1.82)	< 0.0001 (404.74)	4	3 (1.87)a	< 0.0001 (296.45)	9	7 (1.84)	< 0.0001 (701.19)
	Abdomen	640	215 (97.73)		363	157 (98.13)		1003	372 (97.90)	
	Pelvic and abdomen	3	1 (0.45)					3	1 (0.26)	
Affected body organs of infected animals (*N* = 380)										
Single organ infected (goat: *N* = 187; sheep = 147; both = 334)	Gall bladder	34	12 (5.45)	< 0.0001 (717.68)				34	12 (3.16)	
	Kidney	2	1 (0.45)					2	1 (0.26)	
	Large intestine	5	4 (1.82)		2	2 (1.25)		7	6 (1.58)	
	Liver	12	7 (3.18)		10	3 (1.89)		22	10 (2.63)	
	Lung				1	1 (0.63)		1	1 (0.26)	
	Omentum	365	145 (65.91)		276	128 (80.00)		641	273 (71.84)	
	Rumen				2	1 (0.63)		2	1 (0.26)	
	Small intestine	31	13 (5.91)		14	9 (5.63)		45	22 (5.79)	
	Urinary bladder	6	5 (2.27)		4	3 (1.87)		10	8 (2.11)	
Two organs infected. (goat: *N *= 31; sheep = 12; both = 43)	Omentum and liver	61	15 (6.82)	< 0.0001 (39.44)	24	6 (3.75)	0.040 (10.00)	85	21 (5.53)	< 0.0001 (68.44)
	Omentum and lung	2	2 (0.91)					2	2 (0.53)	
	Omentum and urinary bladder	26	6 (2.73)		2	1 (0.63)		28	7 (1.84)	
	Omentum and large intestine	9	1 (0.45)		9	1 (0.63)		18	2 (0.53)	
	Omentum and small intestine	47	4 (1.82)		17	3 (1.87)		64	7 (1.84)	
	Liver and urinary bladder	9	2 (0.91)					9	2 (0.53)	
	Omentum and diaphragm	4	1 (0.45)					4	1 (0.26)	
	Liver and small intestine				3	1 (0.63)		3	1 (0.26)	
Three organs infected	Omentum, liver and urinary bladder	23	1 (0.45)					23	1 (0.26)	
	Omentum, liver and small intestine				3	1 (0.63)		3	1 (0.26)	
Four organs infected	Omentum, small intestine, liver, diaphragm, uterus	8	1 (0.45)					8	1 (0.26)	

The metacestode, *C. tenuicollis*, was found in the abdominal and pelvic cavities and also attached to the omentum, small intestine, liver, gall bladder and urinary bladder of small ruminants (Table 3). The odds of the cyst being detected in the abdominal cavity were significantly higher than in the pelvic cavity (OR = 2477.79; 95% CI: 889.45–6902.46; *p* < 0.0001, *χ* ^2^ = 701.19), while the omentum harboured more cysts compared to all organs put together (OR = 20.03; 95% CI: 13.53–29.66; *p* < 0.0001, *χ* ^2^ = 269.13). Double organ combinations involving omentum were significantly higher (OR = 6.67; 95% CI: 2.60–17.12; *p* < 0.0001, *χ* ^2^ = 16.79) compared to all other combinations put together. Similar trends and observations were recorded when sheep and goats were considered separately (Table 3).

Among the infected animals, the detection of cysts on a single organ was significantly higher in sheep (OR = 1.61; 95% CI: 1.05–2.49; *p* = 0.03, *χ* ^2^ = 4.71) than in goats, while the detection of cysts on four organ combinations was significantly higher in goats (OR = 4.33; 95% CI: 1.46–12.84); *p* = 0.03, *χ* ^2^ = 4.71) than in sheep. Also, infected slaughtered sheep and goats presented an average of 2 (range: 1–11) and 3 (range: 1–25) cysts of *C. tenuicollis*, respectively, in the present study.

## 4 Discussion

Livestock, including small ruminants, was integral to the socio‐economic, cultural and religious activities for most of the communities in the study area. Close human–livestock and dog–livestock interactions were also common, and livestock owners placed great importance on their animals. The small ruminant market in Bamenda municipality is the largest in the Northwest region of Cameroon and provides the live animals for mutton and goat meat requirements to about one million inhabitants of the city, peri‐urban areas and its neighbouring villages (rural areas). Small ruminants slaughtered at the slabs of Bamenda municipality were mainly of the Djallonke type and originated from within the administrative divisions of the Northwest region. With the exception of a few parts (e.g., horn and hooves), almost all other parts and organs of a carcass are edible in Cameroon if passed at slaughter and meat inspection (MINEPIA 2000; Awah‐Ndukum et al. 2012). Prior to the study period, documented information on *C. tenuicollis* in sheep and goats in Cameroon and particularly in the Northwest region was sparse.

The present study showed that *C. tenuicollis* is endemic and widespread in sheep and goats in the study region, and the observed prevalence rate (34.36%) was higher than the prevalence of *C. tenuicollis* in pigs and previously reported prevalence rates in sheep and goats (19.2% (15.1%–24.1%) in Northern Cameroon (Assana et al. 2010), Saudi Arabia (0.45%) (Bakhraibah and Alsulami 2018), India (4.22%–4.83%) (Singh et al. 2015), Tanzania (23.3%) (Miran et al. 2017) and in Baghdad (28.0%) (Al‐Sudani and Al‐Amery 2022). Nevertheless, the finding is lower than the prevalence of *T. hydatigena* cysticercosis (infection rate > 40%) in sheep and goats in Benoue in the Northern Region of Cameroon (Djonmaïla 2016), 56.8% in Central Oromia, Ethiopia (Wondimu et al. 2011) and 72.38% in Dessie, Ethiopia (Gessese et al. 2014). However, the findings are similar to that reported in Ethiopia (34.8%) (Tolossa et al. 2019). The observed prevalence in this study could be due to the husbandry practices with an abundance of stray dogs living in association and sharing the same livestock environments. Also, increases in the prevalence and risk of exposure of domestic animals to *C. tenuicollis* infection in traditional systems of raising animals (extensive or semi‐intensive grazing) have been described (Bayu et al. 2013; Morais et al. 2017; Tolossa et al. 2019). The present study agrees with reports in other endemic countries of Africa where small ruminants often share the same environment with dogs (Gessese et al. 2014; Ouchene‐Khelifi and Ouchene 2017; Yalelet et al. 2018; Addy et al. 2021).

The prevalence of *C. tenuicollis* infection in sheep and goats following ingestion of plants, soil, grass, water and vegetables contaminated by *T. hydatigena* proglottids or eggs passed in dog faeces has been recorded (Miran et al. 2017; Yalelet et al. 2018; Khouloud et al. 2019). Abattoirs and other public waste and garbage disposal sites in the administrative divisions of the present study are usually poorly managed and constantly frequented by stray and scavenging dogs and livestock, including small ruminants. Furthermore, insufficient and inappropriate anthelmintic treatment of animals together with the presence of a large population of stray dogs with easy access to infected offal at open slaughtering sites and garbage were associated with the spread and maintenance of *T. hydatigena* taeniasis/cysticercosis in the region and the high prevalence of *C. tenuicollis* in sheep and goats observed in the study areas. The contributing roles of inappropriate dumping of waste on the high prevalence of *C. tenuicollis* have been previously demonstrated by Abdulatif et al. (2015), Tolossa et al. (2019), Khan et al. (2020) and Addy et al. (2021).

In this study, no significant difference in individual prevalence of *C. tenuicollis* was recorded for goats and sheep. This was associated with similar grazing behaviour and mixed‐husbandry management systems prevailing in the local areas (Senlik 2008; Fube 2015; Ensieh et al. 2020) and high levels of close interactions between small ruminants and dogs, including stray (Awah‐Ndukum et al. 2004; Mohammed and Kadir 2020) and owned dogs. This finding was similar to reports in Turkey (Senlik 2008), Ethiopia (Mekuria et al. 2013) and Ghana (Addy et al. 2021), which stated that there was no significant difference in the prevalence of *C. tenuicollis* in goats and sheep. Though small ruminant (sheep and goats) farmers in rural agropastoral areas of Cameroon use dogs to fight off predators from attacking their livestock, their dogs usually receive little or no medical attention for diseases such as gastrointestinal parasitism, which is widely prevalent in the country (Awah‐Ndukum 2003; Mpoame et al. 2003; Awah‐Ndukum et al. 2007). Contamination of pasture with infected *T. hydatigena* eggs predominantly released by stray and scavenging domestic dogs (Nyero et al. 2015; Asmare et al. 2016; Djiatche 2017; Khouloud et al. 2019; Khan et al. 2020) and cysticercosis in sheep and goats from *T. hydatigena* dog‐infected areas (Djonmaïla 2016; Awe 2017) have been previously described. Clandestine and unmonitored slaughtering of domestic small ruminants, uncontrolled disposal of viscera and trimmings, and poor hygiene practices with livestock waste inappropriately dumped (such as in streams, rivers, open fields, garbage areas, etc.) and the presence of scavenging and stray dogs, which are major factors for higher prevalence of *C. tenuicollis* infestation (Nyero et al. 2015; Tolossa et al. 2019; Khan et al. 2020; Mohammed and Kadir 2020; Wakid and Alsulami 2022), are also common in the study region. Small ruminants are mostly reared in traditional systems (extensive or semi‐intensive grazing) and depend on natural pasture in the study area. However, successful control of cysticercosis in sheep herds has been achieved by including the control of the infection in the definitive host in extensive husbandry systems and preventing potential final hosts from entering intensive livestock systems (Abdollahi et al. 2023). Regular administration of anthelmintic and preventing ingestion of the carcasses of infected small ruminants is essential in controlling the infection in the definitive host (Taylor et al. 2016; Abdollahi et al. 2023).

Overall, there was no difference in the prevalence of *C. tenuicollis* infection according to species and the sex, age and BSC groups of all the animals (goats and sheep) in the present study. However, seasons, weight and location of origin of the animals significantly influenced the prevalence of infected animals (sheep and goats). Also, the prevalence rates were significantly influenced by the location of origin, weight and BSC, as well as the physiological (pregnant and lactating) status of the female goats and age for female sheep. Similarly, Yalelet et al. (2018) and Wakid and Alsulami (2022) had observed that the infection rates were not influenced by domestic small species, sex, age and BSCs among the species. Comparable infection rates previously documented among sheep and goats include 23.09% for sheep and 20.98% for goats (Gessese et al. 2014); 10.80% for sheep and 12.20% for goats (Wondimu et al. 2011); and 12.36% for sheep and 21.37% for goats (Dyab et al. 2017).

Wide ranges of *C. tenuicollis* infection rates were recorded in sheep and goats throughout the study period, ranging from 7.14% to 57.32% for both sheep and goats, 0% to 56.10% for sheep and 7.14% to 65.63% for goats. Higher detection rates were recorded during the wet season than during the dry season, with several fluctuating peaks observed, particularly during the months of May, July, September and December. The seasonal variations were associated with the diversity in ecological conditions (Al‐Sudani and Al‐Amery 2022); epidemiological survival time and dispersion of the eggs (Hailu et al. 2019; Jansen et al. 2021); seasonal dynamics of *C. tenuicollis* (Felefl and Laban 2020) and climate change (Bryson et al. 2020), which could have increased the spread and burden of the parasitism. However, the role of increased livestock movement across open unfenced paddocks with close interactions (inter‐ and intra‐) between animals and environment during transhumance periods (January to March) is not understood.

The omentum was the predominant predilection site for the attachment of *C. tenuicollis* cysts in the present study, followed by the liver and mesentery of the large intestines. Numerous studies (Samuel and Zewde 2010; Gessese et al. 2014; Dyab et al. 2017; Miran et al. 2017; Mokhtaria et al. 2018; Morais et al. 2017; Tolossa et al. 2019; Mohammed and Kadir 2020) had reported that the omentum was the main location for attachment of *C. tenuicollis* cysts. Though the omentum covers a large surface area in the peritoneal cavity (abdominal region) and plays vital immunological roles as a regulator and regional immune responses (Bryson et al. 2020; Meza‐Perez and Randall 2021), the extreme preference of *C. tenuicollis* for the omentum compared to other organs needs to be further investigated. The relative abundance of the cysts on the liver and mesentery may be due to their anatomical location in the abdominal cavity and communication with the omentum. Though the predominant site of attachment of the cysts was on the omentum (< 85%) compared to the other organs (single or all put together), various combinations of affected organs comprising the omentum, liver, small intestines and/or urinary bladder were also noted. In the present study, the relative abundance of *C. tenuicollis* cysts in infected slaughtered sheep and goats showed a similar trend to reports in Tunisia by Khouloud et al. (2019) that recorded cyst intensities of 1.97 and 2.85 in sheep and goats, respectively.

## 5 Conclusion

The study reports on the epidemiological aspects of *C. tenuicollis* and confirmed that it causes health and production problems in sheep and goats in the Northwest region of Cameroon. The study showed widespread distribution and associated factors of the metacestode *C. tenuicollis* in sheep and goats in the region. *C. tenuicollis* cyst was prevalent (34.36%) in all divisions in the region and detected during the entire study period, and no major difference was observed between the rate in goats (35.89%) and sheep (32.45%). Weight, BSC, and origin of the animals, as well as pregnancy and lactating status of female animals and season were the major factors in goats and only age in sheep. *C. tenuicollis* cysts were predominant in the abdominal cavity and mainly attached to the omentum compared to the pelvic cavity and other organs, respectively. Though the study suggests that cysticercosis in small ruminants is neglected, further parasitological, molecular and impact studies would be necessary to determine control measures and socio‐economic impact of the disease in sheep and goat production in the country.

## Author Contributions

**Prudentia Yensi Lawan**: conceptualization, methodology, data curation, investigation, validation, formal analysis, funding acquisition, writing–original draft, writing–review and editing, resources, software, project administration. **Aziwo Tatanja Niba**: conceptualization, validation, formal analysis, supervision, visualization, project administration, writing–review and editing, methodology. **Julius Awah‐Ndukum**: conceptualization, methodology, software, investigation, validation, formal analysis, supervision, project administration, writing–review and editing.

## Ethics Statement

Risk assessments of the project were performed by the researchers to avoid hazards to all persons and animals involved in the study. Permission for the study and ethical approval were obtained from the required authorities in Cameroon [Ministry of Livestock, Fisheries and Animal Industries (MINEPIA) Ref No: MINEPIA/DREPIA/NW/40/335 of 21/09/2022] before carrying out the study. The purpose of the study was explained to the targeted participants (traders and butchers at the small ruminant markets and slaughter slabs in the present study) usually with the assistance of resident veterinarians, local leaders and or trusted intermediaries. An animal was included in the study after an informed verbal consent was given by the owner or trader–butcher. Apart from procedural restraining manipulations for safety purposes, the animals used in the present study were not subjected to suffering. Slaughtering and dressing of sheep and goat carcasses were done following standard procedures as described by the Cameroon veterinary services (MINEPIA 2000) and supported by manuals on routine meat inspection (FAO 1994; Grist 2011).

## Conflicts of Interest

The authors declare no conflicts of interests.

### Peer Review

The peer review history for this article is available at https://www.webofscience.com/api/gateway/wos/peer‐review/10.1002/vms3.70307

## Data Availability

The data used in this study are available from the corresponding author on reasonable request.
